# Pedigree-Free Estimates of Heritability in the Wild: Promising Prospects for Selfing Populations

**DOI:** 10.1371/journal.pone.0066983

**Published:** 2013-06-25

**Authors:** Laurene Gay, Mathieu Siol, Joelle Ronfort

**Affiliations:** 1 Diversity and Adaptation of Mediterranean Species, UMR AGAP 1334, Montpellier, France; 2 Genetics and Ecophysiology of Legume Species, UMR Agroecology 1347, Dijon, France; University of Konstanz, Germany

## Abstract

Estimating the genetic variance available for traits informs us about a population’s ability to evolve in response to novel selective challenges. In selfing species, theory predicts a loss of genetic diversity that could lead to an evolutionary dead-end, but empirical support remains scarce. Genetic variability in a trait is estimated by correlating the phenotypic resemblance with the proportion of the genome that two relatives share identical by descent (‘realized relatedness’). The latter is traditionally predicted from pedigrees (Φ*_A_*: expected value) but can also be estimated using molecular markers (average number of alleles shared). Nevertheless, evolutionary biologists, unlike animal breeders, remain cautious about using marker-based relatedness coefficients to study complex phenotypic traits in populations. In this paper, we review published results comparing five different pedigree-free methods and use simulations to test individual-based models (hereafter called animal models) using marker-based relatedness coefficients, with a special focus on the influence of mating systems. Our literature review confirms that Ritland’s regression method is unreliable, but suggests that animal models with marker-based estimates of relatedness and genomic selection are promising and that more testing is required. Our simulations show that using molecular markers instead of pedigrees in animal models seriously worsens the estimation of heritability in outcrossing populations, unless a very large number of loci is available. In selfing populations the results are less biased. More generally, populations with high identity disequilibrium (consanguineous or bottlenecked populations) could be propitious for using marker-based animal models, but are also more likely to deviate from the standard assumptions of quantitative genetics models (non-additive variance).

## Introduction

The genetic variance available for a trait in a population informs us about its potential ability to evolve in response to novel selective challenges [1]. This corresponds to one of Brookfield’s definitions of the evolvability of a population: “a description of its current standing crop of genetic variability, and the consequence of the extent and nature of this variation for the population’s ability to respond to current selective pressures” [2]. Additive genetic variance can be estimated from the resemblance between relatives by relating the phenotypic covariance of a quantitative trait with the proportion of the genome for which two relatives share genes identical by descent [1], [3]. To achieve this, one would ideally like to know the actual proportion of loci controlling the trait that are identical by descent. This ‘realised relatedness’ is the outcome of a stochastic process (due to Mendelian segregation and linkage) with a variance that depends on genome size [4]–[6]. However, because causal loci are unknown, we traditionally use the expected value of identity by descent given the ancestry [7], [8]. It can be deduced from a pedigree (hereafter called Φ*_A_*), either in an experiment using specific relatedness classes (e.g. full sib-half sib design) or in a population with pedigree data ranging over several generations [9]. However, in wild populations, pedigree information is generally not available except for a few long term studies [10]–[12]. An alternative solution is to estimate the genome-wide average of the realised relatedness between individuals using molecular markers [13].

With the ongoing rise of next generation sequencing, high density SNP panels become available and the realised proportion of the genome that two individuals share identical by descent can be estimated with increasing accuracy [14]. Several estimators of kinship (sometimes called coancestry) and relatedness (or relationship) coefficients have been proposed [14]–[21] and compared [16], [22]–[24]. From these reviews, it appears that the relative performance of each method depends on the set of loci used, on allele frequency distributions, in particular minor allele frequency spectrum [25] and on the average relatedness between individuals in the population. Provided we can estimate it precisely, using the genome-wide average of the realised relatedness rather than resorting to its expected value (Φ*_A_*) could improve the estimation of evolutionarily relevant parameters for quantitative traits (genetic variance, genetic correlations or selection gradients) [7].

Different methods are available to estimate quantitative genetic parameters from molecular marker data (hereafter called pedigree-free methods) [26]. Their respective reliability and suitability depending on the marker used and/or the population genetic structure remain unclear. While such methods based on genome-wide molecular information have received substantial attention by animal breeders and human biologists [27], [28], evolutionary biologists remain very cautious about how useful marker-based relatedness coefficients could be for studying complex phenotypic traits in populations lacking pedigree information [29].

Being able to accurately estimate the genetic variance of a trait is particularly important for inbred or selfing species that have been described as evolutionary dead ends due to the potential loss of genetic diversity [30], [31]. Self-fertilization is common in angiosperms [32], [33] and also occurs in hermaphrodite animals at a lower frequency [34]. Reduced genetic variation in highly selfing populations is frequently observed using molecular markers [35]–[37]. It can be explained by reduced effective population sizes accompanying increased homozygosity [38], enhanced genetic hitchhiking with selective sweeps and background selection caused by a reduced effective recombination rate [39], [40] and frequent bottlenecks following the recurrent extinction - recolonisation events [41]. However, the effect of selfing on the genetic variation relevant for most adaptive change, i.e. quantitative genetic variation, is less clear. For a population with constant allelic frequencies, inbreeding is expected to increase the genetic variance of a trait to the point that when inbreeding is complete, the genetic variance in the population as a whole is doubled and appears as the between-lines component [1]. Simultaneously, inbreeding should reduce quantitative variation due to the fixation of alleles and to a reduced efficiency of selection in maintaining variants (see [42] and [43] for a review of theoretical arguments). Simulations have shown that if non-additive effects are important (dominance or epistasis), additive genetic variance increases with inbreeding, reaching a maximum for intermediate inbreeding coefficients (*F*) and then a declining towards zero at *F = *1 [44], [45]. In agreement with these theoretical expectations, some evidence for reduced within population genetic variance in highly inbreeding populations compared to outcrossing populations has been found [43], [46]. More data are needed to compare the level and components of quantitative genetic variation between selfers and outcrossers and to further understand the consequences of self-fertilization on the adaptive potential of natural populations.

Besides direct effects on genetic variability, selfing generates a correlation in heterozygosity and/or homozygosity across loci, called identity disequilibrium [47]. This will broadly influence both the relatedness between individuals and the variance in relatedness in the population [48], [49] and could improve marker-based estimates of the expected proportion of identity by descent [48]. Thereby, selfing may provide favourable conditions for pedigree-free quantitative genetics.

In this paper we review published results comparing several pedigree-free methods used to estimate quantitative genetics parameters for complex phenotypic traits in wild populations. We then report results from simulations aimed at further comparing the performance of individual-based models (hereafter called animal models) using pairwise relatedness predicted from the pedigree *versus* molecular markers, with a special focus on how mating systems affects the efficiency of these methods.

## Materials and Methods

### Review

As a preliminary step, we searched the literature (using keywords in Web of Science) looking for studies comparing methods for estimating quantitative genetic parameters using molecular markers, for example a pedigree-based animal model and another method, or a simulation study. Our review is based on the classification published by Garant & Kruuk [26], who identified three categories of methods that rely on molecular markers. (1) The ‘Ritland’ method estimates heritability as the covariance between pairwise phenotypic similarity and pairwise relatedness [50]. Alternatively (2), using a maximum likelihood approach, individuals can be classified into known classes of relatedness (for example sibs vs. unrelated) and analyzed in a mixture model [51]. Sibling groups can also be identified within one generation and analyzed in a classic quantitative genetics framework (analysis of variance) or using a more complex model (animal model, [52]). Finally (3), parentage assignment methods can help reconstruct a complete pedigree spanning several generations [13], [53] and quantitative genetics parameters are then derived from an animal model [54]. We expanded Garant & Kruuk’s classification to include two additional methods that are currently available: (4) an animal model method [3], [12], [55] directly using the full pairwise relatedness matrix estimated using molecular markers [56], [57] and (5) a multilocus association method derived from genomic selection [27]. This last method was originally aimed at predicting individual breeding values using molecular markers, in order to accelerate and improve the response to artificial selection. Technically, it relies on multiple regressions with shrinkage, where the phenotype is explained by a set of markers (e.g. [58]).

In order to compare additive genetic variances among traits or taxa, it is common practice to scale it with the total phenotypic variance (heritability) or with the trait mean (coefficient of additive genetic variation). As proposed by Houle [59] and recently confirmed by Hansen *et al.* [60] the additive genetic coefficient of variation is a better predictor of a population’s ability to respond to selection and can be viewed as an accurate measure of evolvability. Nevertheless, heritability remains the most commonly reported measure of evolutionary potential (in particular in all studies using the Ritland method). In our review, we therefore compared heritabilities rather than coefficients of additive genetic variation. This can be misleading in the presence of a positive correlation between the additive variance and other components of phenotypic variance [60]. We also used heritabilities in our simulations in order to remain consistent. We argue that it is not problematic in our simulations as there is no inherent correlation between variance components.

The performance of a statistical inference can be evaluated by the bias, defined as E(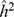 – *h^2^*), where 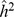 is the estimator and *h^2^* the parameter and the sampling error, E(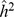 –E(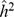))^2^. When reviewing the literature for pedigree- free methods, we would ideally like to compare the bias and sampling error of heritability estimates obtained using pedigrees (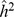 *_ped_*) or one of the marker-based methods (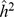 *_marker_*). However, the review of empirical results only provides us with single estimates 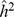 *_ped_* and 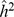 *_marker_* to compare and we cannot say anything about the extent to which either or both are biased and which has smaller sampling error. We therefore only tested whether 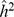 *_ped_* and 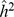 *_marker_* significantly differed for each of the marker-based methods using linear models and also compared their standard errors. Most studies considered several traits that would be wrongly considered as independent. We took this into account by adding a random effect for study. For simulation studies, we report the bias and standard error of the estimators 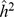 *_ped_* or 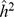 *_marker_*. Again, we tested for an influence of the method used (pedigree or marker-based methods) on the bias using linear mixed models with study as a random effect followed by posthoc tests (with Bonferroni correction) to identify significant pairwise differences. Studies using genomic selection methods generally evaluate the accuracy of the model as the correlation between the estimated breeding value and the real breeding value. We report these accuracies.

We tested whether the absolute value of the bias increases with heritability (measured by pedigree methods or simulated) and assessed the relationship between the bias and the number of markers available (or simulated) using a mixed linear model for each method, with study as a random effect.

### Simulations

We simulate a population of constant size *N* = 500 individuals evolving for a number of generations. Individuals are diploid and unrelated at generation 0 (but they can share alleles identical by state). Generations are non-overlapping. We simulate the genotype of each individual at *L_M_*+*L_QTL_* unlinked loci. The *L_QTL_* loci are causal loci whereas the *L_M_* loci are biallelic non-coding marker loci. Initial allele frequencies are drawn from the distribution expected at mutation – drift equilibrium using a Dirichlet distribution [61]. The phenotype of each individual is controlled by 500 loci (*L_QTL_*) with five alleles each. Allelic effects are randomly drawn from a normal distribution for each allele. The phenotype of an individual is the sum of the two allelic effects at each of the *L_QTL_* loci (*α_i,j_* is the allelic effect of allele *i* at locus *j*) plus a random factor (*ε_ij_*) drawn from a normal distribution representing the environmental effect:
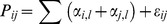
(1)


The variances of the distribution of allelic and environmental effects are adjusted to simulate heritabilities of 0.15, 0.3 and 0.6. The next generation is built either by random mating of pairs of individuals or with a set proportion of selfing (*S* = 0.9) to simulate inbred populations. The simulation program is written in C++ and runs in batch using a custom python script, with 20 replicates per parameter set. The code is available as supplementary material (Zipfile S1).

### Estimation of Genetic Variance Using a Marker-based Animal Model–influence of Mating Regime and Manipulations on Relatedness Matrices

We simulated populations evolving for 10 generations and performed analyses at generation 10, using pedigree and marker-based animal models (method 4 in the review section). Every generation, the pedigree information (mother and father of each individual) was recorded and used to calculate the relatedness coefficient (Φ*_A_*) between pairs of individuals. Genotypes at the *L_M_* marker loci were used to estimate pairwise relatedness using the coefficient introduced by Loiselle *et al.* [20] (thereafter named *K_i,j_*). It does not assume Hardy-Weinberg equilibrium and performs well, even in the presence of rare alleles [24], [62]. To estimate heritability, we fitted a very simple linear model to the simulated phenotype: *y = Za+e* where *y* is the phenotype, *Z* is a design matrix and *a* the vector of additive genetic effects; *e* is the vector of residual effect. The pedigree or the marker loci information were then used to specify a variance–covariance structure for the vector of additive genetic effects *a*, shaped as 2.Φ*_A_*.*σ_A_^2^* when using an animal model including the pedigree or as 2.*K_i,j_.σ_A_^2^* when replacing the Φ*_A_* matrix by a marker-based relatedness matrix. For selfing populations, we used 2.Φ*_A_*.*σ_A_^2^*/(1+ *F*) and 2.*K_i,j_.σ_A_^2^*/(1+ *F*) to account for inbreeding [62], where *F* was the average inbreeding coefficient. *F* was either estimated using pedigrees or approximated as *S*/(2–*S*) [63] for the marker-based method, where the pedigree is supposed unknown. We preliminarily ascertained that both values were highly similar (*F = *0.82 for *S = *0.9). We used restricted maximum likelihood to estimate the additive genetic variance and the standard errors with the program ASReml v3.0 [64]. We examined the effect of the number of marker loci by letting *L_M_* vary between 384, 1500, 3000 or 5000 SNPs. We also manipulated the marker-based relatedness matrix to summarize the information and examined how this influenced the bias in genetic variance estimates. It has become common practice in association studies (GWAS) to truncate the marker-based relatedness matrix by replacing any negative value by zero. We transformed our matrix accordingly.

As in the review section for simulation studies, we measured the bias in heritability as E(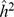 – *h^2^*). We estimated the precision of the estimation using sampling errors, defined as E(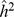 – E(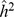))^2^. We compared the bias and sampling errors in heritability estimators (pedigree versus molecular markers) using Wilcoxon signed rank tests. We tested for the effect of the mating regime, trait heritability and number of markers on the bias in heritability estimates using linear models.

The identity disequilibrium created by consanguineous matings could improve marker-based estimates of the expected proportion of identity by descent [48]. We verified this in our simulations by estimating the relatedness at the causal variants (500 QTLs) and examined how accurately it is predicted by the relatedness at the *L_M_* marker loci, in selfing or outcrossing populations, using linear regressions. A slope close to one would indicate that the set of observed SNPs accurately predicts the relatedness at causal loci. Any deviation to one could be caused either by sampling error (due to the limited number of observed SNPs) or by rare alleles in the causal variants. All analyses were run in R version 2.15.1.

## Results

### Review of Published Results

**Figure 1:**
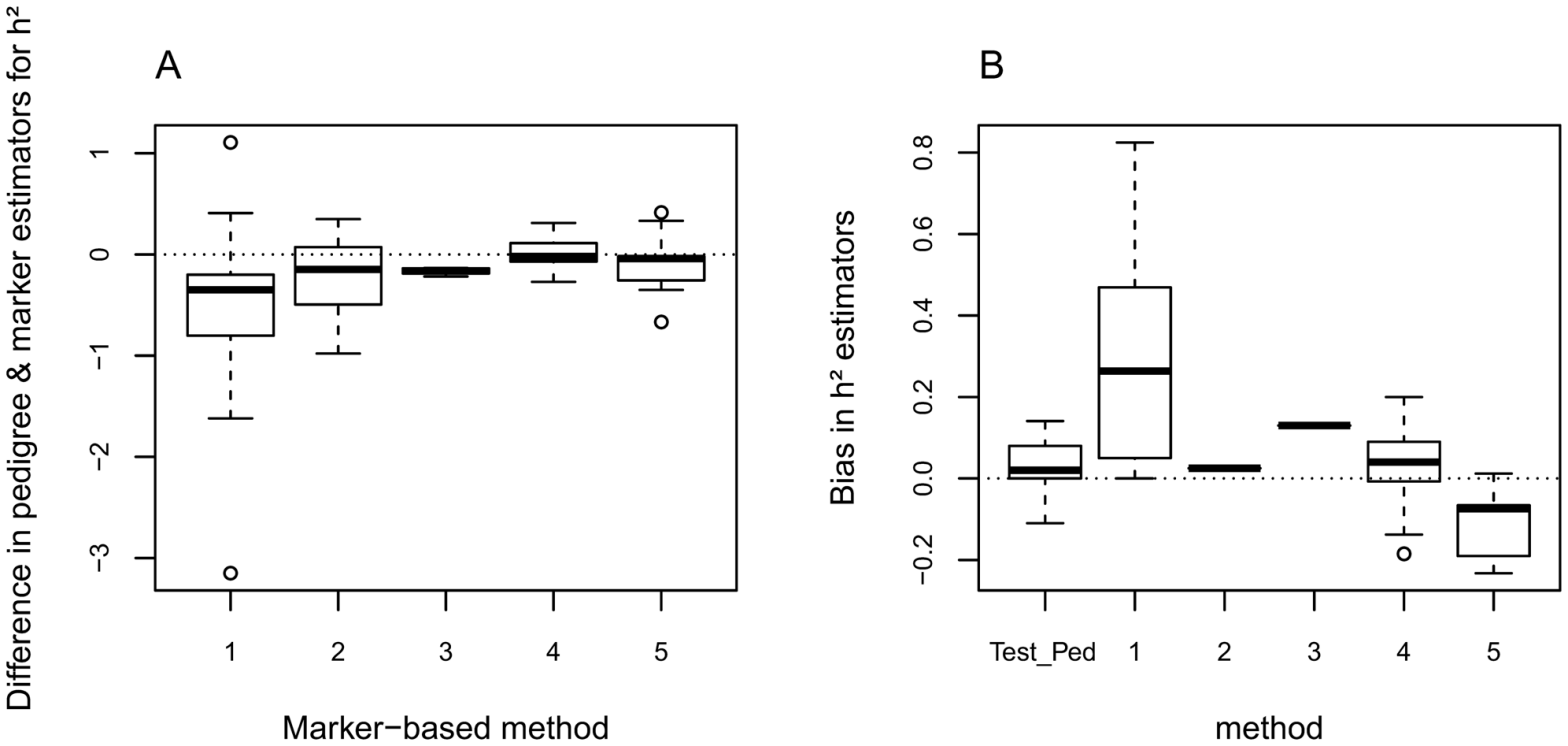
Accuracy of five different marker-based methods to estimate heritability– review of empirical and simulation studies. The efficiency was assessed in a review of 24 empirical studies (A) or 15 simulation studies (B), comparing heritability estimates using pedigree or one of the following methods: 1 - Ritland; 2 - relatedness classes; 3 - reconstructed pedigrees; 4 - marker-based animal model or 5 - genomic selection. Details of the number of studies for each method are given in Table 1. The bias was measured as 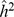 *_marker_* - 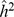 *_pedigree_* in A and as E(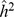 – *h^2^*) in B, where *h^2^* is the simulated parameter. The horizontal line shows the median bias for each method. The bottom and top of the box show the 25th and 75th percentiles. The vertical dashed lines show the maximum and minimum biases and the circles are outliers.

We collected 39 papers comparing heritability estimates based on molecular data or pedigree information. Among those, 24 reported empirical results (Table 1) while 15 were based on simulations only (Table 2). Only seven studies reported heritability estimates for plant species, 16 for animals and one for a protist. Using linear mixed models, we found a significant difference between pedigree-based estimates and marker-based estimates for the Ritland method (χ^2^ = 22.2; *p = *2.5×10^−06^), the relatedness classes (χ^2^ = 5.9; *p* = 0.015) and the reconstructed pedigrees (χ^2^ = 29.8; *p* = 4.7×10^−08^) but not for the animal model (χ^2^ = 0.04; *p = *0.836) or genomic selection (χ^2^ = 0.001; *p* = 0.977). As shown by Figure 1.A, the difference between the heritability estimated using pedigree or molecular markers was lowest with method 4 (marker-based animal model, see also Figure S1). When a dataset was analyzed using Ritland’s method and an animal model in parallel, the latter gave results closest to the estimates obtained using the pedigree [65]. In addition, pedigree-free methods seem to improve the precision of the estimation, except for the Ritland method and the genomic selection method (with Ritland: standard error increased by +0.23; p = 0.062 and +0.13 with genomic selection; p = 0.001).

**Table 1:** Summary of studies comparing estimates of quantitative genetics parameters using pedigree-free methods.

Species	Method^1^	Marker^2^	Individuals	Traits	Kinship coef^3^	Bias^4^	Note	Variance in relatedness	Mating regime^5^	Ref
Bighorn sheep *Ovis canadensis*	1	32 SSR	110 to 150	15	LR - QG - W	−0.20	–	0.0093	Out	[99]
Yellow monkey-flower *Mimulus gattatus*	1	8 allozymes	665	7	R	−0.67	–	0.009 or 0.027 to 0.044 [100]	Mix	[101]
Turkey oak *Quercus laevis*	1	5 SSR	200	1	R	–	no variance of relatedness	0.0007	C+Out	[102]
Soay sheep *Ovis aries*	1	11 SSR	529	6	LR	−0.56	–	0.0005	Out	[79]
Capricorn silvereye *Zosterops lateralis chlorocephalus*	1	11 SSR	479	6	QG	−0.24	–	−0.001 to 0.008	Out	[65]
Shea tree *Vitellaria paradoxa*	1	12 SSR	200	5	LR - W	−0.70	–	0.004	Out	[103]
Yellow box *Eucalyptus melliodora*	1	6 SSR	259	1	LR	*−0.21*	bias relative to Doran and Matheson [104]	0.003	Mix	[105]
Algarrobo Blanco *Prosopis alba*	1	6 SSR	142	13	R	−0.84	–		Out	[106]
Monterey Pine *Pinus radiata*	1	8 SSR	355	1	R	0.41	–	0.009	Out	[107]
Japanese flounder *Paralichthys olivaceus*	1	7 SSR	134	20	LR - QG - R - W	*0.29*	bias relative to method3	0.0001	Out	[108]
Rainbow trout *Oncorhynchus mykiss*	1	16 SSR	628	3	R	*1.70*	bias relative to method3	0.002	Out	[109]
Yellow monkey-flower *Mimulus gattatus*	2	8 allozymes	665	7	–	−0.60	–		Mix	[101]
Salmon *Oncorhynchus tshawytscha*	2	1SLP^6^+1 SSR	170	2	–	0.27	bias relative to Heath *et al.* [110]		Out	[51]
Soay sheep *Ovis aries*	2	12	529	6	–	0.02	–		Out	[79]
Rainbow trout *Oncorhynchus mykiss*	2	16	628	3	–	−0.07	bias relative to method3		Out	[109]
Soay sheep *Ovis aries*	3	12	529	6	CERVUS	−0.17	–		Out	[79]
Rainbow trout *Oncorhynchus mykiss*	3	16	628	3	MCMC	–	no comparison		Out	[109]
Japanese flounder *Paralichthys olivaceus*	3	7 SSR	50 to 134	20	COLONY	–	no comparison		Out	[108]
Common sole *Solea solea*	3	10 SSR	2000	1	PAPA	–	no comparison	0.04	Out	[111]
Cattle - Angus	4	9323 SNP	379	1	SA	0.19	–		Out	[112]
Capricorn silvereye *Zosterops lateralis chlorocephalus*	4	11 SSR	479	6	QG	0.02	–		Out	[65]
Malaria parasite *Plasmodium falciparum*	4	335 SSR	185	8	SA	0.03	–		Mix	[113]
Common sole *Solea solea*	4	10 SSR	2000	1	SA	−0.12	bias relative to method3		Out	[111]
Mouse	4	10000 SNP	2200	4	IBS(i,j)	0.06	–		Out	[67]
Holstein cows	4	54001 SNP	517	3	GRM	−0.10	–		Out	[114]
Angus steers	4	41028 SNP	698	3	GRM	< −0.01	–		Out	[115]
Wheat	5	1279 SNP	599 lines	3	–	0.36	–		Self	[58]
Human	5	294831 SNP	3925	4	–	−0.30	–		Out	[25]
Mouse	5	10 656 SNP	1885	1	–	*0.55*	*Correlation with simulated h^2^*		Out	[116]
Holstein cows	5	54001 SNP	1200	1	–	*0.62*	*Correlation with simulated h^2^*		Out	[117]
Mouse	5	10946 SNP	1884	4	–	−0.31	–		Out	[73]
Mouse	5	10000 SNP	2200	4	–	0.10	–		Out	[67]
Dairy cattle	5	47152 SNP	5217	6	–	−0.04	–		Out	[68]

1 Method: 1 = Ritland’s method; 2 = maximum likelihood on relatedness classes; 3 = sibship or pedigree reconstruction; 4 = animal model with marker-based relatedness matrix; 5 = genomic selection.

2 SSR = microsatellite; SNP = single nucleotide substitution.

3 Method used to estimate pairwise kinship coefficients using molecular markers: LR = Lynch & Ritland [16]; QG = Queller & Goodnight [15]; W = Wang [17]; R = Ritland [21]; SA = % of shared alleles [118]; GRM = genomic relationship matrix [119]; IBS(i,j) = probability of identity by state [120]. For method 3, this column indicates the method of pedigree reconstruction: MCMC (maximizes the pairwise likelihood ratios of being full siblings or unrelated [121]); CERVUS [122]; COLONY [123]; PAPA [124].

4 The bias is defined as the difference between heritability estimated from the pedigree (Φ*_A_*) and heritability estimated using molecular markers in one of the five methods presented 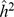 *_marker_*–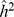 *_ped_*. When no information was available on 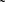 *_ped_* estimated using pedigrees, the bias was measured relative to 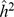 estimated using method 3 (pedigree reconstruction), as indicated in the column “Note”. When several traits were analyzed (column “traits”), we present the average bias.

5 Mating regime: “Out” stands for outcrossing, “Mix” for mixed mating, “Self” for predominantly selfing and “C” for clonality.

6 Single Locus Probe.

**Table 2:** Summary of simulation studies comparing estimates of quantitative genetics parameters using pedigree-free methods.

Method^1^	h^2^	Markers	Individuals	Kinship coef^2^	Bias	Ref
1	0.25	32 SNP	2000 (sib families)	R	0.01	[125]
1	0 to 1	2 to 30	100 to 1000 (sib families)	R	0.05	[126]
1	0.1	10 to 100 - 6 alleles	500	R	0.32	[127]
1	0.5	10 to 100 - 6 alleles	500	R	0.43	[127]
2	0 to 1	2 to 30; 5 to 20 alleles	100 to 1000	–	0.03	[126]
3	0.25	11 SSR	1955	CERVUS - COLONY	0 to 013	[128]
4	0.4 or 0.6	400 SNP	240 inbred lines	L	0.08	[129]
4	0.5	2000 to 5000 SNP	1000	UAR	−0.10	[14]
4	0.33	9000 SNP	1000	SA	0.12	[112]
4	0.33	1000 SNP	1000	SA	0.01	[112]
4	0.1 or 0.3	5 to 100 QTLs	100 (sib families)	SA (on QTLs)	*0.86 (corr)*	[130]
4	0.2	20 to 1200 SNP	20	SA	*0.63 (corr)*	[131]
5	0.8	294831 SNP	3925	–	0.01	[25]
5	0.5	1000 SNP	2200	–	*0.85 (corr)*	[132]
5	0.7/0.3/0.1	6000 SNP	5865	–	*0.89 (corr)*	[116]
5	0.2/0.5/0.9	2000 SNP	1000	–	*0.58 (corr)*	[117]
5	0.1	396 SNP	1000	–	−0.07	[133]
5	0.5	396 SNP	1000	–	−0.20	[133]
5	0.5	5000 SNP	1000 (family structure)	–	*0.79 (corr)*	[134]
ped	0.1		500	pedigree	0.05	[127]
ped	0.5		500	pedigree	0.14	[127]
ped	0.4 or 0.6		240 inbred lines	pedigree	0.03	[129]
ped	0.33		1000	pedigree	−0.11	[112]
ped	0.1 or 0.3		100 (sib families)	pedigree	*0.52 (corr)*	[130]
ped	0.2		20	pedigree	*0.52 (corr)*	[131]

1 The column “Method” is the same as described for Table 1, with the additional category “Ped” that stands for pedigree-based animal models. Numbers in italics indicate that we report the correlation with the simulated heritability (*corr*) rather than the bias.

2 Method used to estimate pairwise kinship coefficients using molecular markers: L = Loiselle [20]; UAR = raw unified additive relationship (estimator of the genome-wide relationship between individuals) [14]; SA = % of shared alleles [135]; pedigree = Φ_A_.

Using the 15 simulation studies, we calculated the bias as E(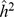
– *h^2^*) (Table 2). The mixed model highlighted a significant effect of the method on the bias (p = 0.005). Posthoc tests showed a significantly higher bias for the Ritland method (average 0.30; *n* = 10) than for the pedigree method (0.04; *n* = 13; p<0.001), the marker-based animal models (0.03; *n* = 15; p = 0.049) or the genomic selection (−0.11; *n* = 12; p = 0.015). There was no significant difference between these three methods (p>0.750). The number of simulation results testing methods 2 and 3 in the dataset we collected was insufficient to compare their biases. The average biases for each method are shown in Figure 1.B. We found no significant difference in standard error between these methods (p = 0.129).

The average value of the bias in heritability tended to increase for traits with low heritabilities (Figure S2.A and S2.B, p<0.0001) and this effect was significant for all methods except the marker-based animal model (p = 0.743). We detected a significant but small negative effect of the number of microsatellites or SNP used on the average value of the bias with the animal model method (p = 0.012 and p = 0.048) (Figure S2.C and S2.D).

### Simulation Results - Heritability Estimation Using a Marker-based Animal Model

**Figure 2:**
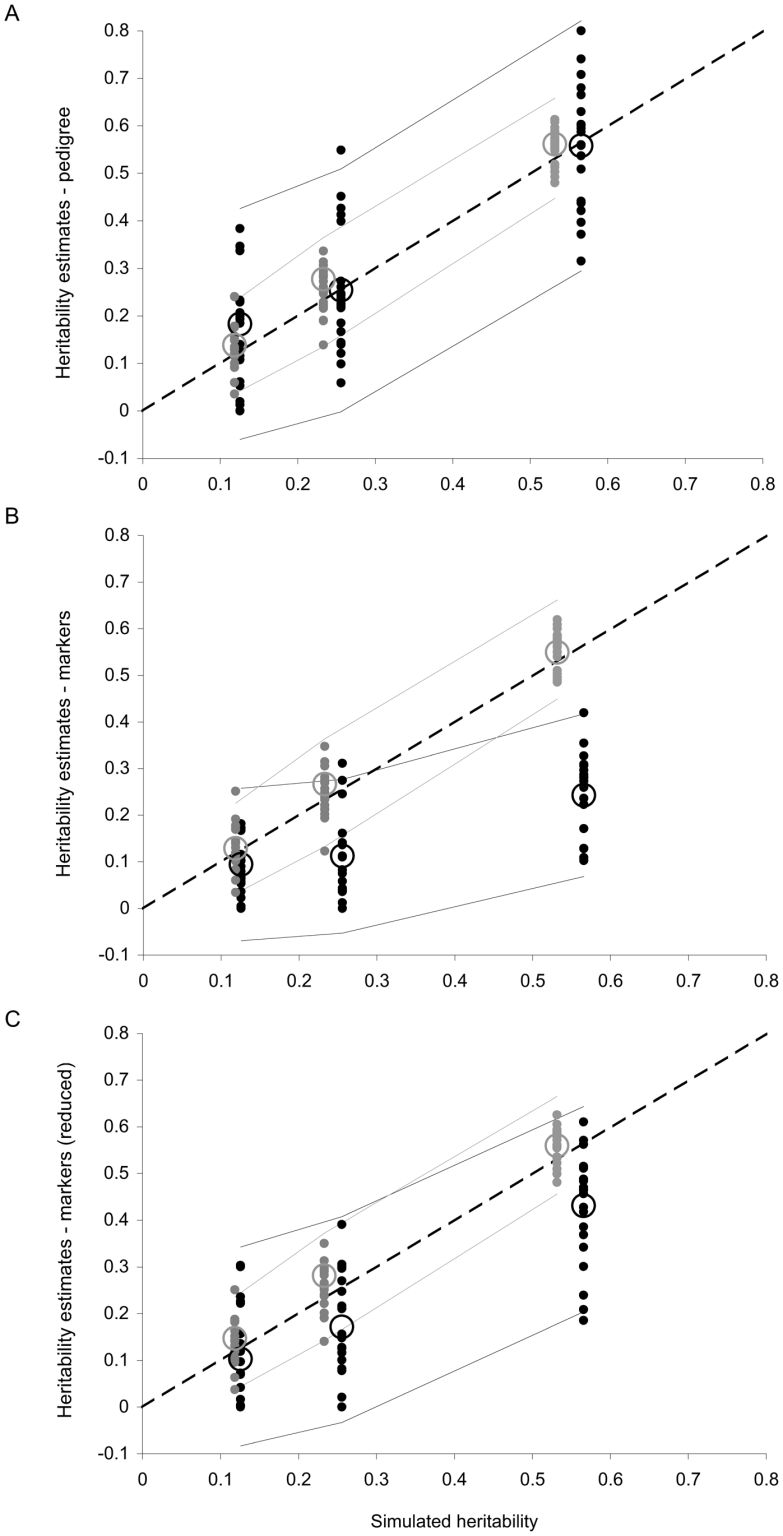
Simulation results testing the accuracy of pedigree or marker-based methods to estimate heritability. This figure shows the correlation between the heritability simulated and heritability estimates obtained using pedigree-based animal models (A), marker-based animal models (B) or marker-based relatedness coefficients truncated before the analysis (C). Each dot stands for a simulated population, with 90% selfing (in grey) or complete outcrossing (in black). Circles stand for means across 20 replicates and solid lines show the 95% confidence intervals, as estimated by Asreml (and averaged across replicates). The dashed lines represent *y = x*.

Simulation results confirmed that using pedigree information in an animal model provides accurate estimates of heritability for both outcrossing and selfing populations, even with low heritabilities (Figure 2.A). We analysed the bias in heritability estimates using Wilcoxon signed rank tests and found that replacing the pedigree-based relatedness matrix by a marker-based relatedness matrix strongly worsens the estimation of heritability for outcrossing populations (average bias = −0.014 with pedigree and 0.174 with markers; *V_Wilcoxon_* = 9; p = 4.1×10^−10^; Figure 2.B) but not for selfing populations (average bias = −0.016 with pedigree and −0.015 with markers; *V_Wilcoxon_* = 738; p = 0.270). The bias in outcrossing populations was reduced when using truncated marker-based relatedness coefficients (0.072; Figure 2.C). Results were the same when testing the effect of the method on the bias using linear models. In addition, if the marker-based method seemed more biased than the pedigree method, the later had higher sampling error (0.15 for the pedigree method and 0.08 for marker-based method) and the difference was significant in outcrossing populations (0.16 versus 0.01; *V_Wilcoxon_* = 1422; p = 4.1×10^−10^) but not in selfing populations (0.14 for both methods; *V_Wilcoxon_* = 1028; p = 0.408).

In outcrossing populations, the absolute value of the bias with the marker-based method increased with higher simulated heritabilities (*F_53,1_* = 155.0; p<10^−16^), but it should be pointed out that with low heritabilities (0.15) the model sometimes failed to converge or estimated an additive variance not significantly different from zero.

**Figure 3:**
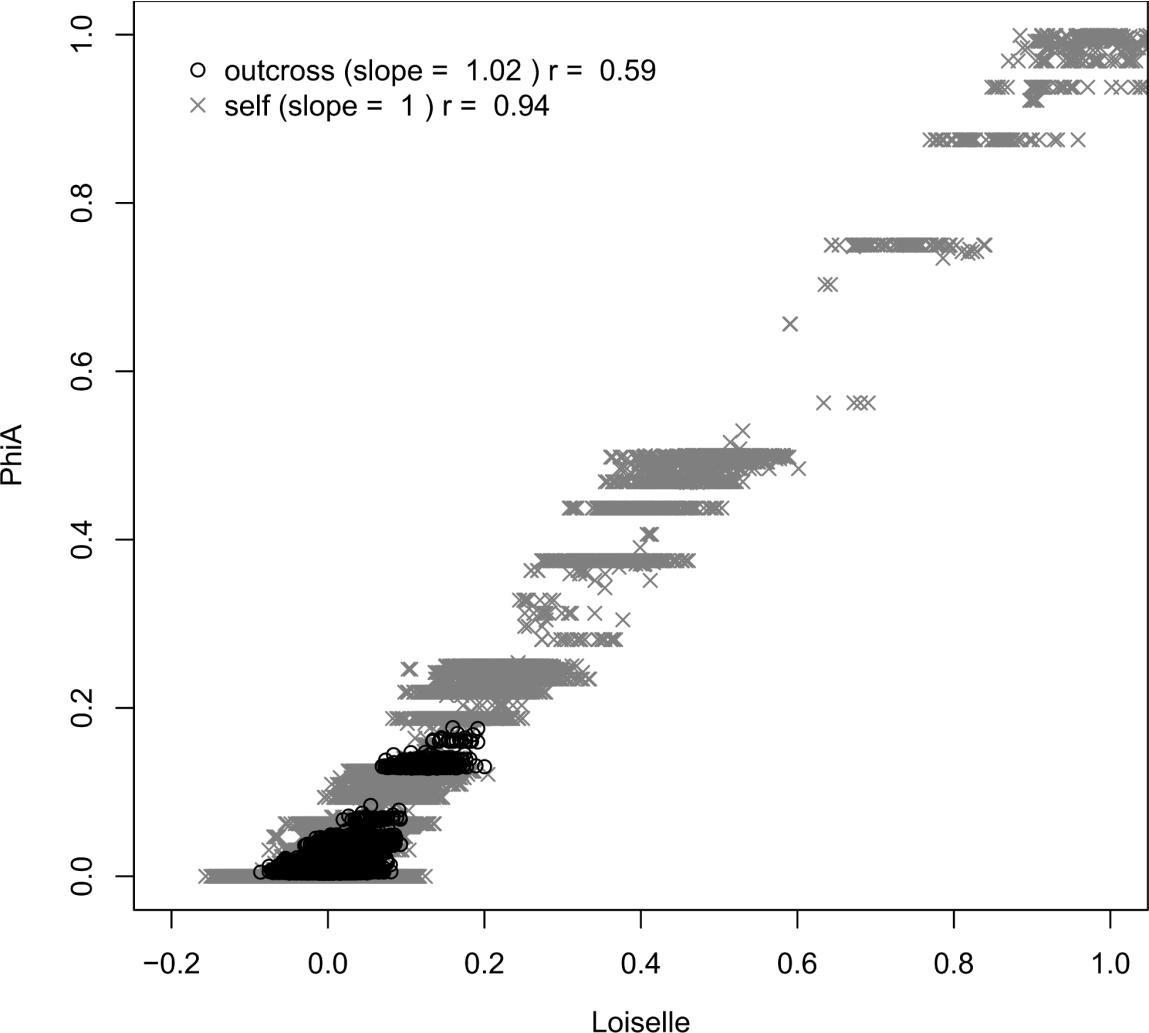
Higher mean and larger variance in pairwise relatedness coefficients in selfing compared to outcrossing populations. Regression between pairwise Loiselle coefficients estimated using 1500 SNP and Φ*_A_*. The population comprised 500 individuals with 90% selfing (grey crosses) or complete outcrossing (black circles). The legend indicates the slope of the regression of Φ*_A_* against Loiselle and the correlation coefficient *r*. The variance in relatedness was 0.0026 in the outcrossing population and 0.0108 in the selfing population (within the range of variances observed in wild populations, see Table 1 and [23]).

**Figure 4:**
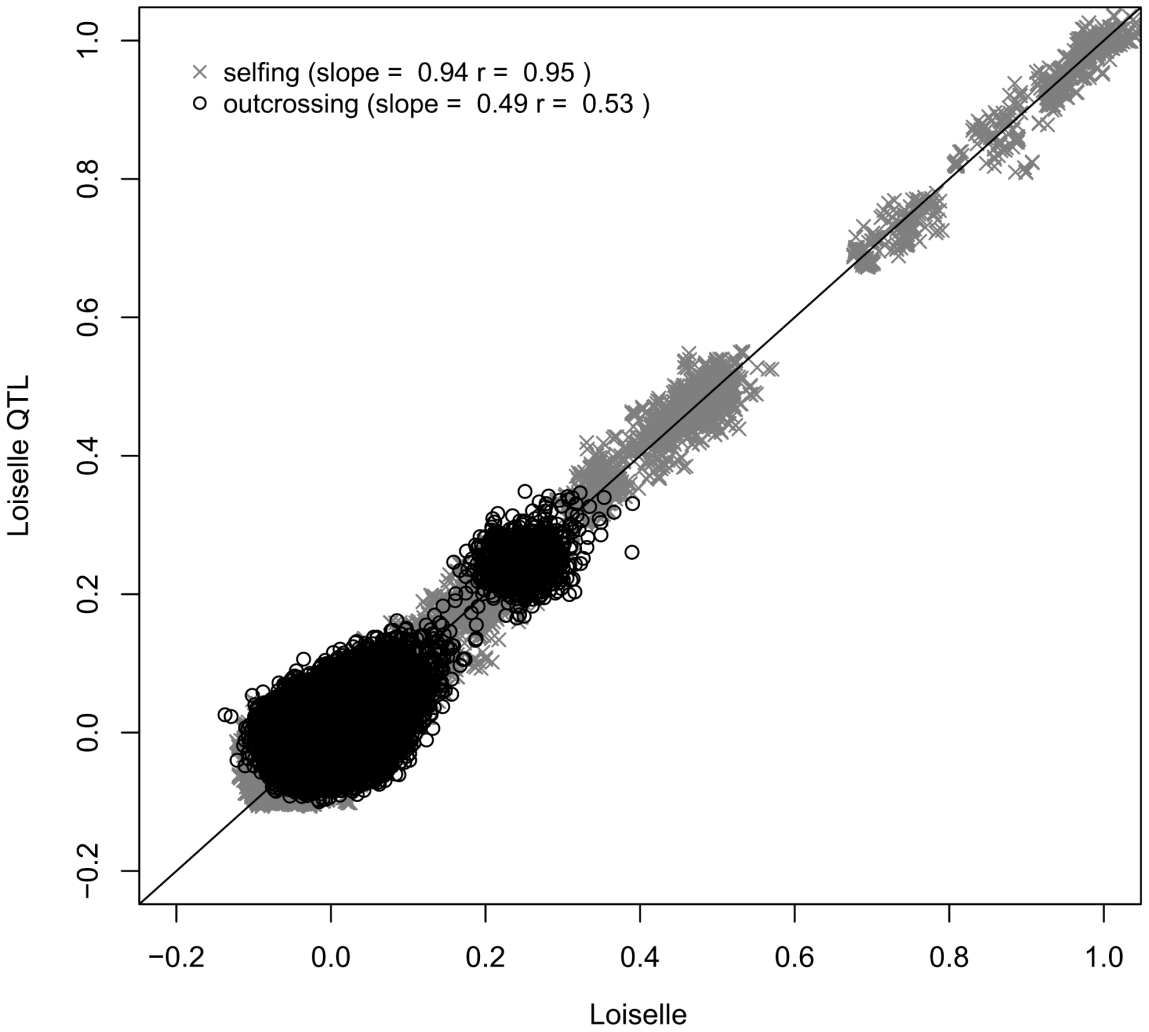
Relatednesses at causal and marker loci are more closely correlated in selfing than in outcrossing populations. Regression between pairwise Loiselle coefficients estimated using 1500 SNPs and pairwise Loiselle coefficients estimated using the allele frequency at QTLs determining the phenotypic trait. Outcrossing populations are shown in black and selfing populations (selfing rate 90%) in grey. The legend indicates the slope of the regression and the correlation coefficient *r*. The slope is expected to be close to one if the relatedness at causal loci is accurately predicted by the relatedness at observed SNPs.

Selfing decreased the bias in marker-based heritability estimates. With a heritability of 0.3, for example, the bias was more than seven times larger in outcrossing compared to selfing populations (*F_36,1_* = 57.2; p = 6.10^−9^). The distribution of pairwise relatedness in selfing populations may explain such a better performance in estimating heritability [48], [49]. Indeed, as shown on Figure 3, pairwise relatedness coefficients have a higher mean and a larger variance in selfing compared to outcrossing populations. This effect extends beyond the simple influence of population size (Figure S3). The higher performance of the marker based method under selfing can also be related to the fact that the relatedness at causal loci is more closely correlated with the relatedness at a set of marker loci in selfing than in outcrossing populations (Figure 4). This is in agreement with results by Szulkin *et al.* [48] on inbreeding coefficients and is caused by high identity disequilibrium in selfing populations.

**Figure 5:**
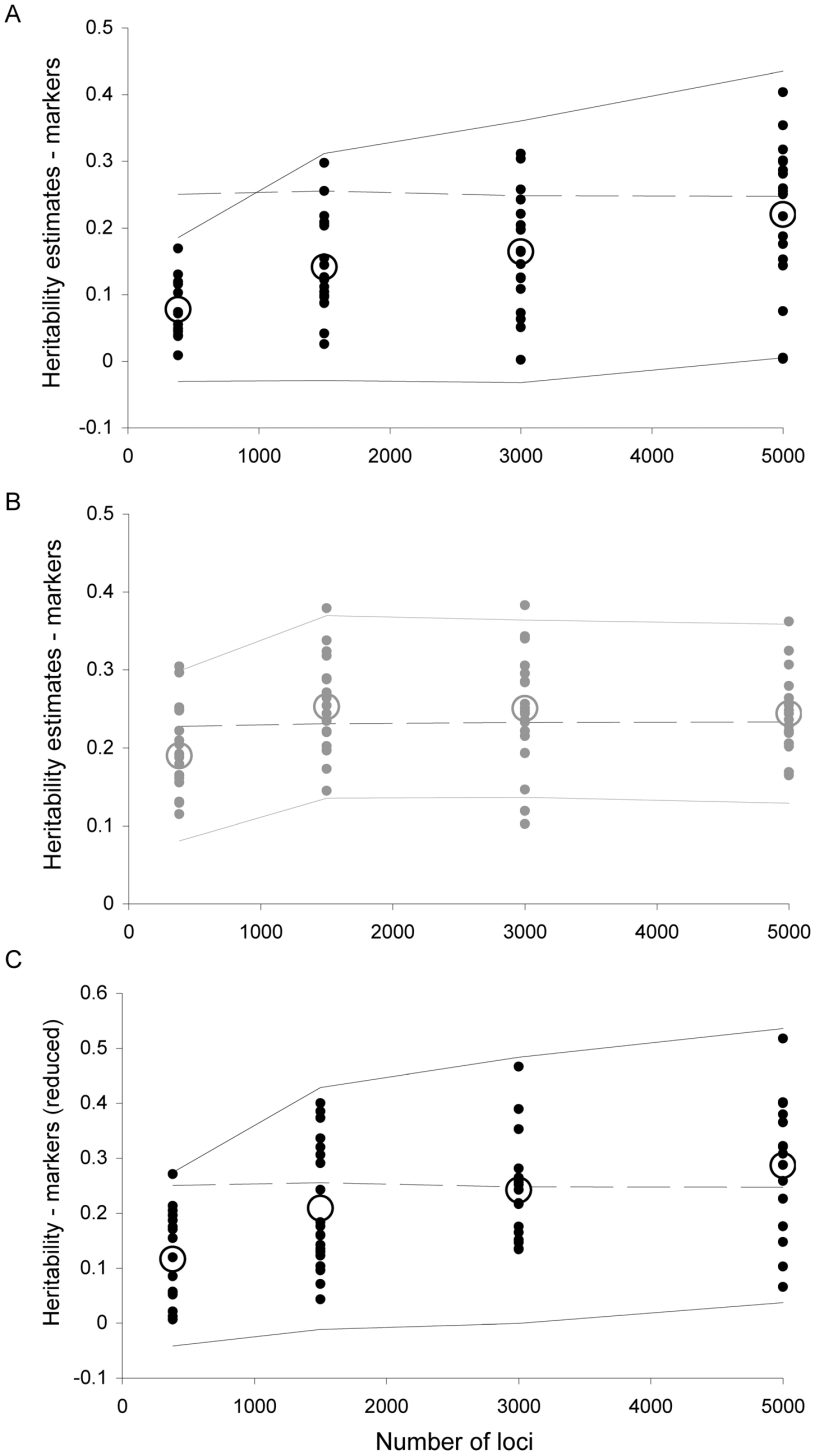
Heritability estimates become more accurate with the number of marker loci used to estimate relatedness. Influence of the number of loci used to estimate pairwise relatedness coefficients (Loiselle coefficients) on the bias in heritability estimates, when using a marker-based animal model. Each dot stands for a simulated population of 500 individuals, with complete outcrossing (panel A, in black) or 90% selfing (panel B, in grey). Panel C shows the results when marker-based relatedness coefficients are truncated before the analysis. Large circles stands for the average heritability over the 20 replicated simulations. The confidence intervals estimated in Asreml for each replicate were averaged over the 20 replicates and are shown as solid lines. The dashed line stands for the simulated heritability.

Figure 5 highlights that when a larger number of loci is used to estimate the matrix of pairwise relatedness, heritability estimates become more accurate, most probably because the prediction of Φ*_A_* is improved. The linear model showed that the absolute value of the bias decreases significantly with the number of markers (*F_78,1_* = 11.9; p = 0.001). If marker-based relatedness coefficients are truncated, the effect becomes non-significant (*F_78,1_* = 0.7; p = 0.389) and a lower number of markers is required to get an accurate estimate of heritability. In selfing populations, there is no relationship between the bias and the number of marker used (*F_78,1_* = 0.8; p = 0.371). 384 markers seem already sufficient to estimate heritability reasonably well. Surprisingly, the sampling error of the estimates did not decrease with the number of loci.

## Discussion

### Pedigree-free Methods to Estimate Quantitative Genetics Parameters in the Wild: What have we Learnt in Nearly 20 Years?

Reviewing empirical and simulation studies of quantitative genetics in wild populations using marker-based estimates of relatedness confirms that it is extremely difficult to derive reliable estimates for quantitative genetic parameters in wild populations using Ritland’s pairwise regression model, as suggested by several authors [26], [65], [66]. Nevertheless, being a pioneer, the Ritland method played a significant role in stimulating the development of further marker-based methods to estimate quantitative genetics parameters in wild populations. Despite performing slightly better than the Ritland method, the relationship classes method (method 2) requires a known family structure with only two classes of relatedness and is therefore of restricted use [52]. Methods 3 and 4 both use the statistical machinery of the animal model (mixed model) after using molecular markers to reconstruct the pedigree (method 3) or the relatedness matrix (method 4). But empirical and simulation results suggest that method 4 performs best. Finally, genomic selection (method 5) has become extremely popular among breeders, and even though their main purpose is to predict breeding values with the highest accuracy, some of these studies report estimates for additive genetic variance [27]. Nevertheless, the lowest biases are found in studies using samples with family structure [67], [68] and simulation studies seem more encouraging than actual empirical results (Tables 1 and 2). Besides, despite using a colossal number of SNPs for genomic selection (and not only those showing significant association with the phenotype – GWAS [69]), the SNPs still often explain only a small proportion of heritability (missing heritability) [25], [70], [71]. Insufficient linkage disequilibrium between causal variants and genotyped SNPs and low minor allele frequency of causal variants might be involved [25]. Currently, models combining pedigree and molecular markers are being developed to partition the additive genetic variance into genomic and “remaining polygenic” components [68], [72], [73]. These results highlight that for traits with highly polygenic determinism, the classic infinitesimal model [74], [75], as implemented in the animal model, might still perform best [27].

### Promising Prospects for Marker-based Animal Models?

Our review showed that animal models including marker-based relatedness matrices (method 4) offer promising prospects. Nevertheless, our simulations show that using molecular markers instead of pedigrees seriously worsens the estimation of quantitative genetics parameters in outcrossing populations, even if an increased number of loci and truncated relatedness coefficients improved the result. Conversely, our simulations in selfing populations suggest that pedigree-free methods are successful. We discuss several arguments that could help explain this contrast, in the light of our simulation results and the literature available about inbreeding.

Firstly, we sampled individuals from a single generation in our simulations (non-overlapping generations, i.e. annual populations). Such a sampling design constrains the variance in pairwise relatedness in outcrossing populations (no parent-offspring relatedness and a probability of 1/*N* to be full-sibs) but not so much in selfing populations (full-sibs from selfing events have Φ*_A_* >0.5). More generally, the variance in relatedness in a population is strongly affected by its size and mating system (see Figure 3, Figure S3 and [49]). Variances in relatedness are generally low in large outcrossing populations (see [23] and the column “variance in relatedness” in Table 1) but this constrains pedigree methods just as much as marker-based methods, and require collecting data over a large number of generations.

A more serious issue is that uncertainty in relatedness estimates is ignored when estimating heritability using this pedigree-free method and this could be a problem if uncertainty is high. Previous studies have shown that marker-based estimates of inbreeding coefficients are improved by non-random mating because it generates identity disequilibrium [48]. We observe the same thing here for pairwise relatednesses (i.e. the inbreeding coefficient of a hypothetical offspring) (Figure 3 and 4). In outcrossing populations, it remains that a very large set of markers is required to estimate the ‘realised relatedness’ without too much uncertainty [16], [22] and to capture the variance within relatedness classes more accurately than when using the pedigree (Φ*_A_*) [4], [5], [28], [69].

It is worth pointing out that in our simulations, we used the complete pedigree (no missing individual), exempt from errors. Even in domestic animals, pedigree errors have been estimated to range between 1% and 10% [76]. In wild populations, the requirement of large sampling efforts and the occurrence of extra-pair paternity will inevitably scale down the pedigree information available for the analysis and might favour the use of alternative methods for a wide range of populations.

Finally, our simulations do not consider non-genetic sources of resemblance between relatives (common environment, maternal effects…). Such non-genetic effects are expected to overestimate heritability quite markedly [77]. Nevertheless, without transplantation or cross-fostering experiment, these effects are difficult to account for with any of the methods currently available (including pedigree methods, see [78]), in particular if there are few different classes of relatedness in the dataset [79].

### Promising Prospects for Selfing Populations

Stebbins [30] suggested that extreme selfing is an “evolutionary dead-end”, because it reduces genetic diversity within populations and may thereby lower their adaptive potential compared to outcrossing populations. Until recently, accurate comparisons of the levels of additive variance between selfers and outcossers were impaired by methodology because most studies on quantitative genetic variation in plants involved analyses of variance between and within families [80], and the pollinations required to derive paternal families were rarely performed (e.g. in 13 out of 37 studies reported by Charlesworth & Charlesworth, [43]), particularly in selfing species. Animal models coupled with marker-based relatedness information offer an inclusive, conceptually simple and flexible framework to quantify additive variance in plant and animal populations by taking advantage of the recombined genotypes produced by rare outcrossing events in their wild environment [69].

Yet, in selfing populations, inbreeding generates additional variance components specifically associated with dominance effects of alleles when autozygous (identical by descent) (detailed in equation (4) in Shaw *et al.* [81]). The covariance between the genotypic values of two individuals X and Y becomes [80], [82]:
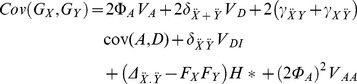
(2)where *V_A_* and *V_D_* are the additive and dominance variances, *cov(A,D)* is the covariance between the additive effect of alleles and their autozygous dominance deviation, *V_DI_* is the total variance due to autozygous dominance effects, H* is the inbreeding depression and *V_AA_* the variance due to additive-additive epistatic effects (other epistatic variances are neglected here). Each variance component is preceded by a probability measure (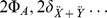), a function of the identity of alleles by descent (described in further details by Cockerham [83] and Harris [82]). This equation highlights the complexity of quantitative genetics in partially inbred populations. In our simulations, we only focused on V_A_ and neglected the effect of directional dominance because we were interested in methods providing comparable estimates of additive variance in selfing and outcrossing populations. These additional variance components could be included in the mixed model (*y = Za +…+ e*), keeping in mind that their estimation is not straightforward without a complex experimental design [84]. Besides, dominance effects due to heterozygote effects (*d_ij_* in Harris [82]) are expected to contribute little to genetic variance in highly selfing populations where homozygosity is very high [80]. Therefore, neglecting V_D_ in selfing populations is not as inaccurate as in outcrossing populations, where it is rarely examined. Theoretical models also predict that inbreeding depression resulting from deleterious recessive alleles should be purged with selfing [85], [86]. Some empirical data support this prediction [87], [88], but the opposite has also been reported [89], in agreement with the prediction that mildly deleterious mutations could accumulate in selfing populations [90]. Finally, with partial selfing the estimate of V_A_ using the animal model can be inflated by variance due to additive-additive epistasis, directly proportional to Φ*_A_* ^2^. Importantly, this is also true for outcrossing species, even if we expect lower effects [91]. We are therefore confident that additive variance estimated using marker-based animal models in selfing populations should be comparable with estimates in outcrossing populations, in spite of inbreeding.

It remains that such estimates of additive variance may not be sufficient to predict the response to selection (*R*) when there is directional dominance and epistasis. Simulations suggest that evolution in partially selfing populations can strongly differ from the predictions obtained using the breeders’ equation (*R = h^2^ S* where *S* is the selection differential), even if the later accounts for inbreeding depression [81]. Methods have been suggested to extend the breeder’s equation to selfing populations [92]–[96], but predictions are difficult when all individuals do not share the same level of inbreeding, as expected in most natural populations.

### Conclusion

Our literature review highlighted that more testing is required for the most promising marker-based methods: animal models including a marker-based matrix of relatedness and genomic selection. Our simulations of the animal model showed that estimates in selfing populations are as accurate when using molecular markers or pedigrees, thanks to their high identity disequilibrium. It is undeniable that a very large set of molecular markers is required in large random mating populations, but recent advances in next generation sequencing technologies provide encouraging prospects, even for non-model species [97], [98]. More generally, populations with high identity disequilibrium (consanguineous or bottlenecked populations) could promote the use of marker-based animal models, but at the same time are more likely to deviate from the standard assumptions of quantitative genetics models (e.g. non-additive variance).

## Supporting Information

Figure S1 **Correlation between heritability estimates obtained using pedigree-based animal models or one of the five marker-based method.** Each dot stands for an empirical result and the colour indicates the method (Ritland in black; relatedness classes in grey; pedigree reconstruction in pink; animal model in green and genomic selection in blue). The dashed lines represent *y = x*.
(DOC)

Figure S2
**Slight increase in the bias with lower heritabilities or lower number of markers.** The panels A and B show the relationship between the bias in heritability estimate and the value of heritability for the empirical data *(*in A – bias expressed as 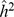 *_marker_* - 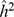 *_pedigree_)* or the simulation data (in B – bias expressed as E(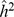 – *h^2^*)). The panels C and D show the relationship between the bias in heritability estimate and the number of microsatellites or SNPs used. As explained in the text, the bias did not systematically increase for traits with low heritabilities but was more variable. Surprisingly, we found no overall relationship between the bias and the number of markers used in empirical data.(DOC)

Figure S3 **Influence of population size and mating regime on the variance in pairwise relatedness in a population.** Selfing could improve marker-based estimation of heritability because it affects the structure and the variance of pairwise relatedness, as has been shown for the inbreeding coefficient. We assessed the effect of the reduced effective population size in selfing populations (*N*e = *N/*(1+ *F*)) by simulating populations with varying census size (*N = *50, 100, 250, 500) and different mating regimes (outcrossing in black and 90% selfing in grey). Error bars stand for the standard error estimated from 10 replicated simulations. This figure confirms that pairwise relatedness coefficients have a higher mean and a larger variance in selfing compared to outcrossing populations and that this effect extends beyond the simple influence of population size (e.g. large excess of variance in a selfed population of *N = *50 compared to an outcrossed population of *N = *100). The identity disequilibrium created by selfing might explain such higher variance in relatedness.
(DOC)

Table S1 **Full data literature review.**
(XLS)

Zipfile S1 **Simulation program in C++.**
(ZIP)
