# Supplementary material for: Pedigree-Free Estimates of Heritability in the Wild: Promising Prospects for Selfing Populations
Source: PLoS One. 2013 Jun 25;8(6):e66983. doi: 10.1371/journal.pone.0066983 (PMC3692515; doi:10.1371/journal.pone.0066983)
Supplement: Figure S1 — Correlation between heritability estimates obtained using pedigree-based animal models or one of the five marker-based method. Each dot stands for an empirical result and the colour indicates the method (Ritland in black; relatedness classes in grey; pedigree reconstruction in pink; animal model in green and genomic selection in blue). The dashed lines represent y = x. (DOC) [file pone.0066983.s001.doc]

**Figure S1**

**Figure S1.** **Correlation between heritability estimates obtained using pedigree-based animal models or one of the five marker-based method.** Each dot stands for an empirical result and the colour indicates the method (Ritland in black; relatedness classes in grey; pedigree reconstruction in pink; animal model in green and genomic selection in blue). The dashed lines represent *y = x*.
