# Supplementary material for: Pedigree-Free Estimates of Heritability in the Wild: Promising Prospects for Selfing Populations
Source: PLoS One. 2013 Jun 25;8(6):e66983. doi: 10.1371/journal.pone.0066983 (PMC3692515; doi:10.1371/journal.pone.0066983)
Supplement: Figure S2 — Slight increase in the bias with lower heritabilities or lower number of markers. The panels A and B show the relationship between the bias in heritability estimate and the value of heritability for the empirical data (in A – bias expressed as marker - pedigree) or the simulation data (in B – bias expressed as E( – h2)). The panels C and D show the relationship between the bias in heritability estimate and the number of microsatellites or SNPs used. As explained in the text, the bias did not systematically increase for traits with low heritabilities but was more variable. Surprisingly, we found no overall relationship between the bias and the number of markers used in empirical data. (DOC) [file pone.0066983.s002.doc]

**Figure S2**

A B

C D

**Figure S2. Slight increase in the bias with lower heritabilities or lower number of markers.** The panels A and B show the relationship between the bias in heritability estimate and the value of heritability for the empirical data *(*in A – bias expressed as *ĥ²_marker_* - *ĥ²_pedigree_)* or the simulation data (in B – bias expressed as E(*ĥ²* – *h²*)). The panels C and D show the relationship between the bias in heritability estimate and the number of microsatellites or SNPs used. As explained in the text, the bias did not systematically increase for traits with low heritabilities but was more variable. Surprisingly, we found no overall relationship between the bias and the number of markers used in empirical data.
