# Supplementary material for: Pedigree-Free Estimates of Heritability in the Wild: Promising Prospects for Selfing Populations
Source: PLoS One. 2013 Jun 25;8(6):e66983. doi: 10.1371/journal.pone.0066983 (PMC3692515; doi:10.1371/journal.pone.0066983)
Supplement: Figure S3 — Influence of population size and mating regime on the variance in pairwise relatedness in a population. Selfing could improve marker-based estimation of heritability because it affects the structure and the variance of pairwise relatedness, as has been shown for the inbreeding coefficient. We assessed the effect of the reduced effective population size in selfing populations (Ne = N/(1+ F)) by simulating populations with varying census size (N = 50, 100, 250, 500) and different mating regimes (outcrossing in black and 90% selfing in grey). Error bars stand for the standard error estimated from 10 replicated simulations. This figure confirms that pairwise relatedness coefficients have a higher mean and a larger variance in selfing compared to outcrossing populations and that this effect extends beyond the simple influence of population size (e.g. large excess of variance in a selfed population of N = 50 compared to an outcrossed population of N = 100). The identity disequilibrium created by selfing might explain such higher variance in relatedness. (DOC) [file pone.0066983.s003.doc]

**Figure S3**

**Figure S3. Influence of population size and mating regime on the variance in pairwise relatedness in a population.** Selfing could improve marker-based estimation of heritability because it affects the structure and the variance of pairwise relatedness, as has been shown for the inbreeding coefficient. We assessed the effect of the reduced effective population size in selfing populations (*N*e = *N* / (1 + *F*)) by simulating populations with varying census size (*N* = 50, 100, 250, 500) and different mating regimes (outcrossing in black and 90% selfing in grey). Error bars stand for the standard error estimated from 10 replicated simulations.

This figure confirms that pairwise relatedness coefficients have a higher mean and a larger variance in selfing compared to outcrossing populations and that this effect extends beyond the simple influence of population size (e.g. large excess of variance in a selfed population of *N* = 50 compared to an outcrossed population of *N* = 100). The identity disequilibrium created by selfing might explain such higher variance in relatedness.
